# The essential inner membrane protein YejM is a metalloenzyme

**DOI:** 10.1038/s41598-020-73660-6

**Published:** 2020-10-20

**Authors:** Uma Gabale, Perla Arianna Peña Palomino, HyunAh Kim, Wenya Chen, Susanne Ressl

**Affiliations:** 1 Department of Molecular and Cellular Biochemistry, Indiana University Bloomington, 212 S Hawthrone Dr, Bloomington, IN 47405 USA; 2 Department of Neuroscience, The University of Texas At Austin, 100 E. 24th St., NHB 2.504, Austin, TX 78712 USA

**Keywords:** Biochemistry, Microbiology, Structural biology, Infectious diseases

## Abstract

Recent recurrent outbreaks of Gram-negative bacteria show the critical need to target essential bacterial mechanisms to fight the increase of antibiotic resistance. Pathogenic Gram-negative bacteria have developed several strategies to protect themselves against the host immune response and antibiotics. One such strategy is to remodel the outer membrane where several genes are involved. *yejM* was discovered as an essential gene in *E. coli* and *S. typhimurium* that plays a critical role in their virulence by changing the outer membrane permeability. How the inner membrane protein YejM with its periplasmic domain changes membrane properties remains unknown. Despite overwhelming structural similarity between the periplasmic domains of two YejM homologues with hydrolases like arylsulfatases, no enzymatic activity has been previously reported for YejM. Our studies reveal an intact active site with bound metal ions in the structure of YejM periplasmic domain. Furthermore, we show that YejM has a phosphatase activity that is dependent on the presence of magnesium ions and is linked to its function of regulating outer membrane properties. Understanding the molecular mechanism by which YejM is involved in outer membrane remodeling will help to identify a new drug target in the fight against the increased antibiotic resistance.

## Introduction

Indiscriminate use of antibiotics has resulted in a crisis of emergence of antibiotic resistant bacterial strains. The World Health Organization supports the larger scientific community in the statement that the world is running out of working antibiotics^1^. Targeting specific mechanisms of antibiotic resistance is especially critical for Gram-negative bacteria such as *Escherichia coli* (*E. coli*) and *Salmonella typhimurium* (*S. typhimurium*) because of the recurrent outbreaks affecting society^2–4^.

Pathogenic bacteria such as *E. coli* and *S. typhimurium* have developed several strategies to protect themselves against the host immune response and antibiotics. These mechanisms create antibiotic resistance allowing Gram-negative bacterial survival and replication^5–7^. The bacterial cell membrane plays a critical role in limiting the effectiveness of antibiotics by acting as a barrier and preventing the diffusion of antibiotics and other harmful chemicals into the cell^8, 9^. Understanding the functional role of proteins and enzymes acting at the interface of the bacterial cell membrane is fundamental to address antibiotic resistance and address the effects of bacterial outbreaks.

The cell envelope of Gram-negative bacteria is made of an inner membrane (IM) and an outer membrane (OM), and the periplasmic space in between that contains a thin layer of peptidoglycan^9^. The OM is an asymmetric lipid bilayer; the inner leaflet is composed of phospholipids and the outer leaflet is made of lipopolysaccharides (LPS). Lipid A (endotoxin) is a component of LPS and a key virulence factor in endotoxin shock^10^. The O-antigen polysaccharide that is linked to LPS is highly antigenic and has a striking flexibility and structural diversity^11^. One strategy employed by Gram-negative bacteria to survive or hide from the host immune system is the modification of the lipid A moiety of LPS. This is facilitated by various inner membrane enzymes such as phosphoethanolamine (PEA) transferases, namely EptA and mobilized colistin resistance (MCR) family of proteins belonging to the alkaline phosphatase superfamily^12–14^. They mediate the decoration of lipid A with PEA resulting in the OM resistance against cationic antimicrobial peptides (CAMPs) and lower affinity to Toll-like receptor 4^7, 12^. Another strategy of OM remodeling is to increase the incorporation of cardiolipin, which is a crucial component of the cytoplasmic membrane and OM that binds bacterial proteins during sporulation and cell division, respectively^15, 16^. Cardiolipin is synthesized at the inner membrane (IM), and cardiolipin trafficking in *Salmonella* to the OM was shown to be controlled by the two-component PhoPQ system^17^. Increased levels of cardiolipin in the OM are found when bacteria experience stress, such as higher temperature (≥ 42 °C)^18^, increased osmotic pressure^19^, in stationary phase^20^, as well as encountering host immune response during pathogenesis^5^.

**Figure 1:**
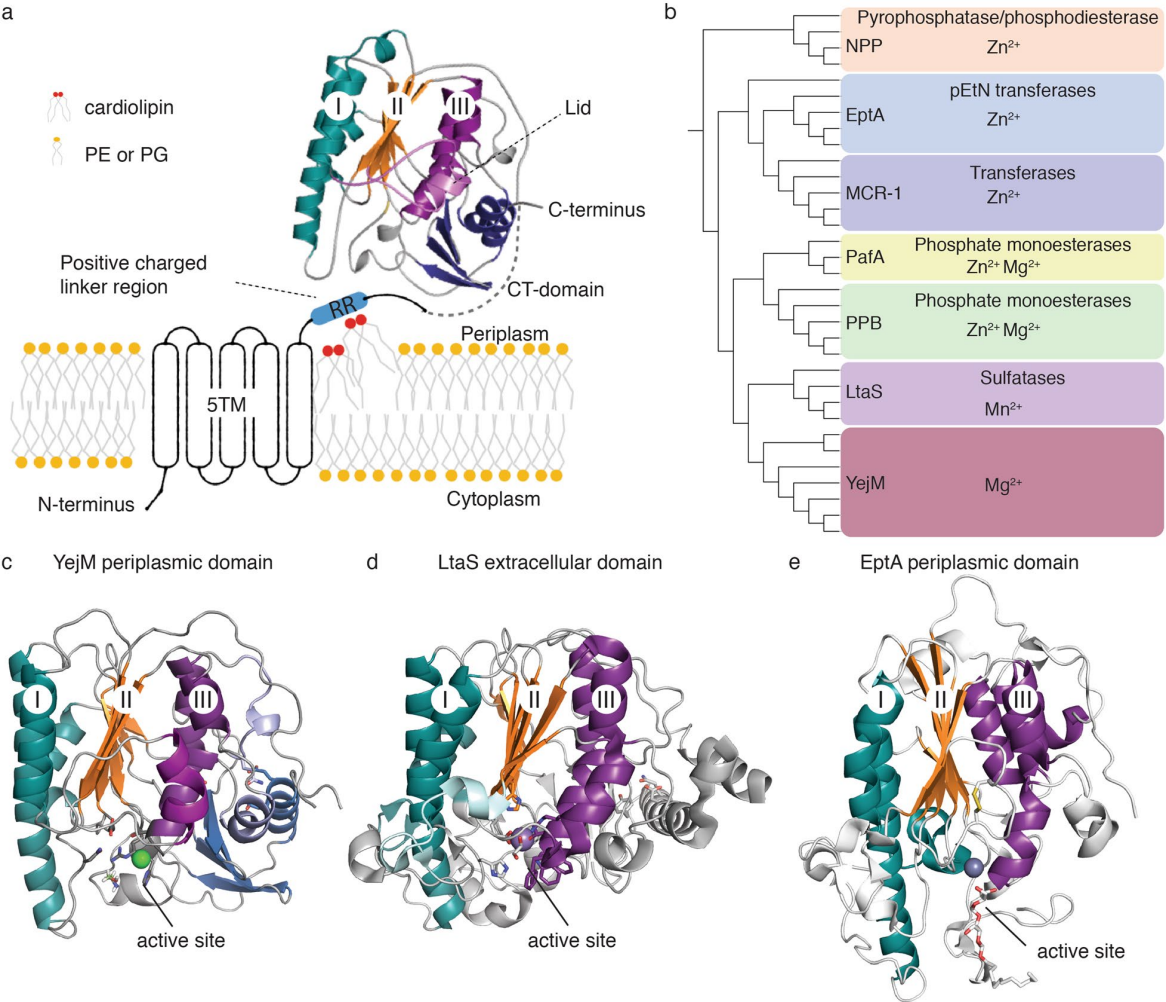
Overview of YejM structure, constructs, and phylogenetic connection of YejM to selected members of the larger hydrolase superfamily. (**a**) Overview of YejM structure. The N-terminal transmembrane domain containing 5 helices (5TM) and linker region with positively charged arginine residues (RR) are illustrated, cardiolipin lipids are indicated to bind to the RR linker region, PE and PG lipids are non-distinguished creating the membrane plane. The periplasmic domain (PD) is presented as our crystal structure of wildtype YejMPD with three layers: I turquoise, II orange, and III deep purple, indicated within the α/β hydrolase core domain. The lid in light purple covers the proposed hydrophobic cardiolipin binding pocket between layers II and III. The C-terminal (CT) dark blue. Sequence between the RR linker region and the beginning of the structure is indicated by a dotted line in light gray. (**b**) Phylogenetic tree of sequences of α/β hydrolase core domain and if present the CT domain only from various members within the hydrolase superfamily. Each clade is indicated by a colored box naming the protein family, their known or predicted enzymatic function, and known metal ions essential for their respective enzymatic functions. (**c**) crystal structure of YejMPD with indicated location of active site. (**d**) LtaS soluble domain with indicated location of active site. (**e**) EptA periplasmic domain with indicated location of active site.

The essential gene *yejM* was discovered in *E. coli* and *S. typhimurium*^21, 22^. The homologue in *S. typhimurium* was termed *pbgA* (PhoPQ-barrier gene A) and proposed to play a crucial role in increased cardiolipin levels in the OM^22^. *yejM* codes for an inner membrane protein with five predicted helical transmembrane domain (5TM), followed by a positively charged arginine rich (RR) linker region and a C-terminal periplasmic domain (PD) (Fig. 1a). Based on its sequence, YejMPD is functionally predicted to be a sulfatase and it is fundamental to OM modifications and immune response. Mutants lacking the PD are viable, whereas deletion of the 5TM domain is lethal for Gram-negative bacteria^21, 22^. Importantly, *E. coli* strain LH530 lacking the YejMPD showed an increased sensitivity to temperature and antibiotics such as vancomycin^21^. However, *E. coli* strains lacking YejM were rescued by overexpression of inactive or active forms of phosphopantetheinyl transferase (AcpT)^21^, that plays a minor role in lipid metabolism of *E. coli* K-12^23, 24^. The YejMPD is also essential for the virulence of *S. typhimurium*; strains lacking YejMPD show no increase of cardiolipin in the OM, have increased permeability under highly PhoPQ activated environment, and fail to survive inside the host cells^22^. More recently YejM has been shown to be involved in other membrane homeostasis pathways. Several recent publications and a crystal structure suggest that YejM regulates LPS biosynthesis by preventing excessive degradation of LpxC, a key enzyme in that pathway^25–29^, and the absence of YejMPD results in increase in lipid A core molecules in IM^25^. LpxC is a zinc-dependent metallo-amidase that performs the first committed step in LPS biosynthesis^30^, and is constantly degraded by the FtsH protease^31^. YejM interferes with LpxC degradation, by interacting with another protein YciM (aka LapB) that presents LpxC to the protease FtsH for degradation^26^. Therefore, YejM plays a crucial role in maintaining LpxC levels, and thus is involved in homeostasis of PL/LPS ratio in the OM that is vital for cell survival. An additional role in the stationary phase was proposed for YejM, by promoting cyclopropane ring formation of phospholipids, especially during stress^25^. This mechanism was found to be bypassed by certain mutations in LpxC and YciM (LapB), thus it must be connected to the mechanism of preventing LpxC degradation. Together, these recent findings shed light on the essentiality of YejM for Gram-negative bacteria, suggesting YejM’s involvement in several membrane remodeling pathways.

Structures of two YejMPD homologues show similarity to hydrolase-fold enzymes like arylsulfatases and alkaline phosphatases^32^. Despite very low sequence similarity, the α/β hydrolase core structure of YejMPD is similar to members of the hydrolase superfamily, including the extracellular domain of lipoteichoic acid synthase (LtaS) of *Staphylococcus aureus*^32^. The soluble domain structure of LtaS revealed a bound Mn^2+^ ion at the enzyme active site near the catalytic nucleophile threonine residue^13^. Structures of PEA transferases like EptA and MCR show the same fold and an active site with bound metal ions and substrates. The conserved and crucial catalytic nucleophilic threonine residue is especially important in catalysis since bacteria showed decrease inhibition by CAMPs, such as colistin and polymyxin, when it is substituted by alanine in MCR-1^33^. A recent crystal structure of YejM/PbgA from *S.* Typhimurium reveals a novel fold for the 5TM region, which shows a deep and long cleft that spans the 5TM and periplasmic domain^34^. Two CL-binding sites were described in the structure, which are both in the membrane-embedded region, and occupy approximately opposite sides of the TM domain. Notably, both CL molecules are located at the perimeter and not inside the cleft of the 5TM domain. The headgroup of one CL molecule in the structure is in direct contact with two arginines, R215 and R216, which have previously been shown to be important for CL binding^22^. The structure revealed a conserved D-X9-H motif in the 5TM domain proposed to be a putative catalytic site that resembles phospholipases^34^. How YejM facilitates outer membrane remodeling whether by aiding cardiolipin translocation or by its involvement in LPS synthesis pathways is unknown.

Here, we report a novel enzymatic function of YejM. We have solved high-resolution crystal structures of wildtype and mutant YejMPD that reveal an intact active site with bound metal ions and highly conserved catalytic nucleophilic threonine residue. We demonstrate that YejM has a phosphatase activity that is dependent on the presence of magnesium ions. Further, we show that the enzymatic activity of YejM depends on the presence of its intact active site and the 5TM domain.

## Results

### Identification of the active site in YejM and structural homologues

We solved crystal structures of the periplasmic domain of YejM (YejM residues 241–586) from *S. typhimurium*: crystal structures of the wildtype YejMPD and the YejMPD alanine mutation at F349 to the resolutions of 2.35 Å and 2.05 Å, respectively (Table 1 and Supplemental Table 1). Their general architecture resembles the α/β hydrolase fold, made up of alternating α-helices and β-sheets forming three layers (Fig. 1a)^32^. The α/β hydrolase core is the landmark domain shared within the large hydrolase superfamily including sulfatases, phospho-, mono-, and diesterases, and metalloenzymes (Fig. 1b; Supplemental Table 2). Compared to other α/β hydrolase-fold enzymes, YejM has an additional C-terminal (CT) domain of unknown function (Fig. 1c). This CT domain shows structural similarity to a few other proteins such as colicin^35^ and kinases such as PLK1 (Supplemental Table 3). Interestingly, in some instances, this domain acts as a site for oligomerization^36^.

**Table 1:** X-ray data collection and refinement statistics for YejMPD structures.

	YejMPD	YejMPD-F349A
PDB ID	6VAT	6VC7
Data collection	ALS BEAMLINE 4.2.2	ALS BEAMLINE 4.2.2
Wavelength (Å)	1.00003	1.00003
Space group	P 32 2 1	P 21 21 21
**Cell dimensions**		
a, b, c (Å)	113.80,113.80,299.78	121.45,125.10,182.75
α, β, γ (°)	90.00, 90.00, 120.00	90.00,90.00,90.00
Resolution (Å)	59.655–2.35 (2.39–2.35)*	47.50–2.05 (2.08–2.05)
Rmerge	0.224 (2.192)	0.112(2.078)
I/σI	11.1 (0.7)	13.8 (0.6)
CC1/2	0.993 (0.212)	0.996 (0.188)
Completeness (%)	99.9 (99.0)	85.88 (48.97)
Redundancy	9.6 (5.0)	6.4 (3.7)
**Refinement**		
Resolution (Å)	56.9—2.35 (2.434—2.35)	43.57—2.05 (2.123—2.05)
No. reflections	94,485 (9231)	150,131 (8464)
Rwork/Rfree	0.1963/0.2489	0.1847/0.2270
No. atoms	17,290	17,703
Protein	16,358	16,353
Ligand/ion	245	340
Water	687	1010
B-factors	52.67	47.21
Protein	53.00	47.01
Ligand/ion	61.84	62.56
Water	41.64	45.18
**R.m.s deviations**		
Bond lengths (Å)	0.004	0.005
Bond angles (°)	0.62	0.77
**Ramachandran statistics**		
Favored (%)	92.11	94.59
Allowed (%)	6.38	4.39
Outliers (%)	1.51	1.02

*Statistics for the highest-resolution shell are shown in parentheses.

Despite their high sequence variability, proteins from the hydrolase superfamily have a conserved metal-specific active site that is located at the base of layers II and III of the hydrolase domain (Fig. 1c–e). Most of these proteins are metalloproteins (Supplemental Tables 2 and 3). YejM has predicted structure and domain similarities with bacterial proteins involved in antibiotic resistance. This includes MCR proteins^14, 33^*,* EptC from *Campylobacter jejuni*^37^, EptA from *Neisseria meningitidis*^12^, etc. (Supplemental Table 3). Unlike YejM and EptA/B/C, which are encoded on the chromosome, the genes encoding MCR1 proteins are located on plasmids and highly transferable across species^14, 38^.

**Figure 2:**
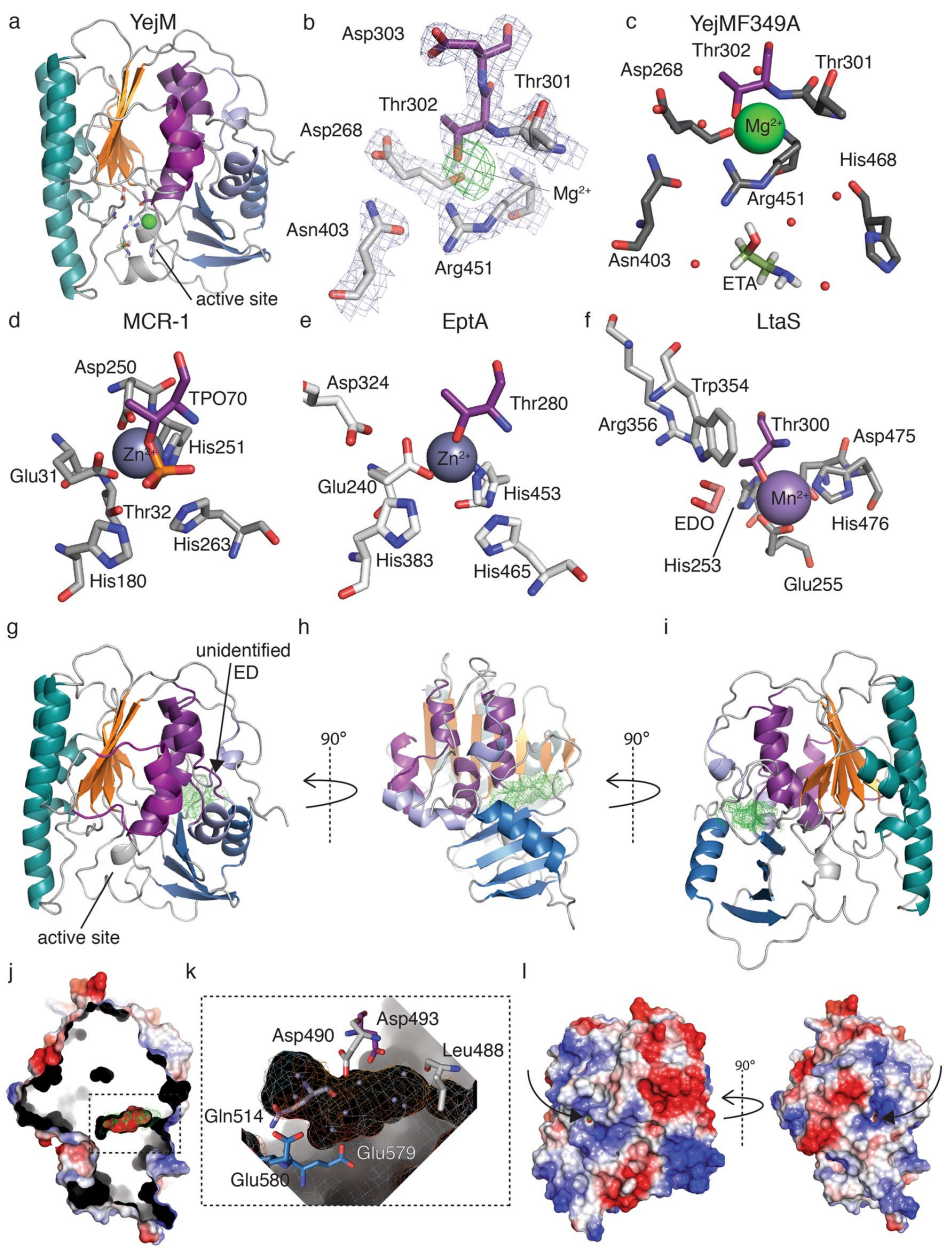
Location of novel metalloenzyme YejM active site and substrate sites**.** (**a**) YejMPD with putative magnesium ion (Mg^2+^) (green) in a larger vestibule at active site below layer II and III. (**b**) Active site of YejMPD-F349A with conserved threonine Thr302 (deep purple) from layer III, putative Mg^2+^ shown by 2Fo-Fc density map (green mesh) at sigma level 2.5, Thr301, Thr302, Asp303, Asp268, Asn403, and Arg451 with 2Fo-Fc electron density (light blue mesh) at sigma level 1.5. (**c**) Active site of YejMPD-F349A with conserved threonine Thr302 (deep purple) from layer III, other residues involved in Mg^2+^ (green sphere) and substrate ethanolamine (ETA) are Asp268, Asn403, Arg451 and His468, prospective specific waters (red spheres). (**d**) Active site of MCR-1 around zinc (Zn^2+^, blue sphere) include conserved phosphorylated threonine TPO70, Glu31, Thr32, His180, Asp250, His251, His263. (**e**) Active site of EptA around Zn^2+^ include conserved threonine Thr280, Glu240, Asp324, His383, His453 and His465. (**f**) Active site of LtaS with conserved threonine Thr300 (deep purple) from layer III involved in coordination of manganese (Mn^2+^, purple sphere) and substrate 1,2-Etheanediol (EDO) in stick representation orange, together with residues Glu255, Asp475, His253, Trp354, Arg356, His476. (**g**) Front view of YejMPD-F349A, black arrow pointing towards the additional density observed in the Fo-Fc map (ED) at 2.2 sigma level (green mesh), located at interface between layer III and the CT domain. (**h**) YejMPD side view. (**i**) YejMPD back view. (**j**) Electrostatic surface presentation of YejMPD side view and cropped in Z plane to show large negatively charged pocket with additional Fo-Fc map (green mesh). (**k**) Zoom in on the negatively charged pocket lined by residues Leu288, Asp490, Asp493, Gln514 and Glu580. Dummy atoms (light blue spheres) placed into the pocket surrounded by the electrostatic surface overlaid in mesh and solid representation. (**l**) Electrostatic surface of YejMPD back and side views. Black arrows points towards positively charged access funnels (port 1) and (port 2) towards the visible Fo-Fc density (green mesh), indicating its accessibility from the outside.

We identified a metal ion binding site in YejM that is conserved across many members of the larger phosphatase super-family (Fig. 1b) and is located at the base of layers II and III (Figs. 1c and 2a–c). Phylogenetic analysis of periplasmic domains only across selected members of the phosphatase super-family revealed that YejM is more closely related to the LtaS in Gram-positive bacteria, and more distantly to PEA transferases EptA and MCR-1 in Gram-negative bacteria (Fig. 1b).

### YejM has an active site similar to other metalloenzymes

Our YejMPD structures show electron densities near the conserved threonine residue Thr302, which could be best assigned with Mg^2+^ during structure building and refinement, suggesting the presence of a metal binding site (Fig. 2b,c). Multiple sequence and secondary-structure based alignments (Clustal Omega^39^, Promals^40^), revealed that the active site residues of LtaS and EptA are structurally aligned with those of YejM (Fig. 2c–f). The metal coordination in our YejMPD structures resembles metal coordination in MCR-1, EptA, and LtaS (Fig. 2d–f). The active site coordination of YejM contains the conserved threonine 302 (Thr302) located at the base of layers II and III. Asp268, Asn403, Arg451 and His468 residues are involved in metal, water and substrate coordination (Fig. 2b,c). We compared the active site coordination and found similarities to: (i) MCR-1 with residues around its Zn^2+^ coordinated by Thr70 that is phosphorylated in this structure to TPO70, and further residues Glu31, Thr32, His180, Asp250, His251, His263^41^ (ii) EptA with residues around its Zn^2+^ ion coordinated by Thr280, and Glu240, Asp324, His383, His453 and His465^12^ (Fig. 2d,e). The active site of YejM also resembles coordination of the active site around the Mn^2+^ of LtaS, with conserved threonine Thr300 and residues Glu255, Asp475, His253, Trp354, Arg356, His476^13^ (Fig. 2f). We conclude that although there are differences between the active sites, most residues and their charges, particularly the catalytically crucial nucleophilic threonine, are highly conserved across the active sites of other metalloenzymes (Supplemental Figs. 1 and 2).

### The active site residues of YejM are highly conserved across homologues

To examine the active site residues conservation across YejM homologues, we chose three sequences centered around Asp268 (metal and substrate binding), Thr302 (catalytic residue and metal binding), and Arg451 (metal binding). In addition, we used secondary structure boundaries of the hydrolase domain to define these three sequences centered around Asp268, Thr302, and Arg451 (Supplemental Fig. 2a). Our phylogenetic analysis revealed very high conservation for the sequence around Asp268 and Thr302, and more sequence flexibility around Arg451. Key residues Asp268, Thr302 and Arg451 show nearly 100% conservation across all homologues. Whereas Asp268 is 100% conserved across YejM homologues, Thr302 is substituted to an alanine in the clade including *Klebsiella pneumoniae* (KLEPN) and *Klebsiella michiganensis* (KLEMI) (Supplemental Fig. 2a). Their sequence retained a threonine at the same place as YejM Thr301, which may compensate the loss of threonine at position Thr302. Two species from the clade comprised of *Enterobacter cloacae* (ENTCL) and *Raoultella terrigena* (RAOTE) indicate a serine substitution at Thr301; serine can also act as a nucleophile to form a covalent phospho-intermediate (Supplemental Fig. 2a). At the position of Arg451 we observed a change to histidine in some species; this conserves the positive charge and characteristic of this site that is likely involved in metal binding (Supplemental Fig. 2a). The major divisions between the clades are defined around conservation at His468. Whereas it is highly conserved amongst *E. coli*, *Salmonella* and *Citrobacter*, *Enterobacter* has an asparagine, and *Klebsiella* and *Raoultella* have a glutamine at this position (Supplemental Fig. 2a,b). YejM homologues have slight differences around the conserved main active site residues and accommodate for various substrates and metal binding. Blasting our three initial *S. typhimurium* sequences (Supplemental Fig. 2a) against the database resulted in several conserved types of sequence alterations around all three active site sequences, building specific signature sequences that we found assembled across species (Supplemental Fig. 1). Furthermore, we found that *E. coli* uses only type 1 and *S. typhimurium* only type 2 sequences, and other homologues used many combinations of various type of sequences (Supplemental Fig. 1), which further increases the accommodation of substrate and metal use in a variety of enzymatic functions.

### YejM is a magnesium specific phosphatase

**Figure 3:**
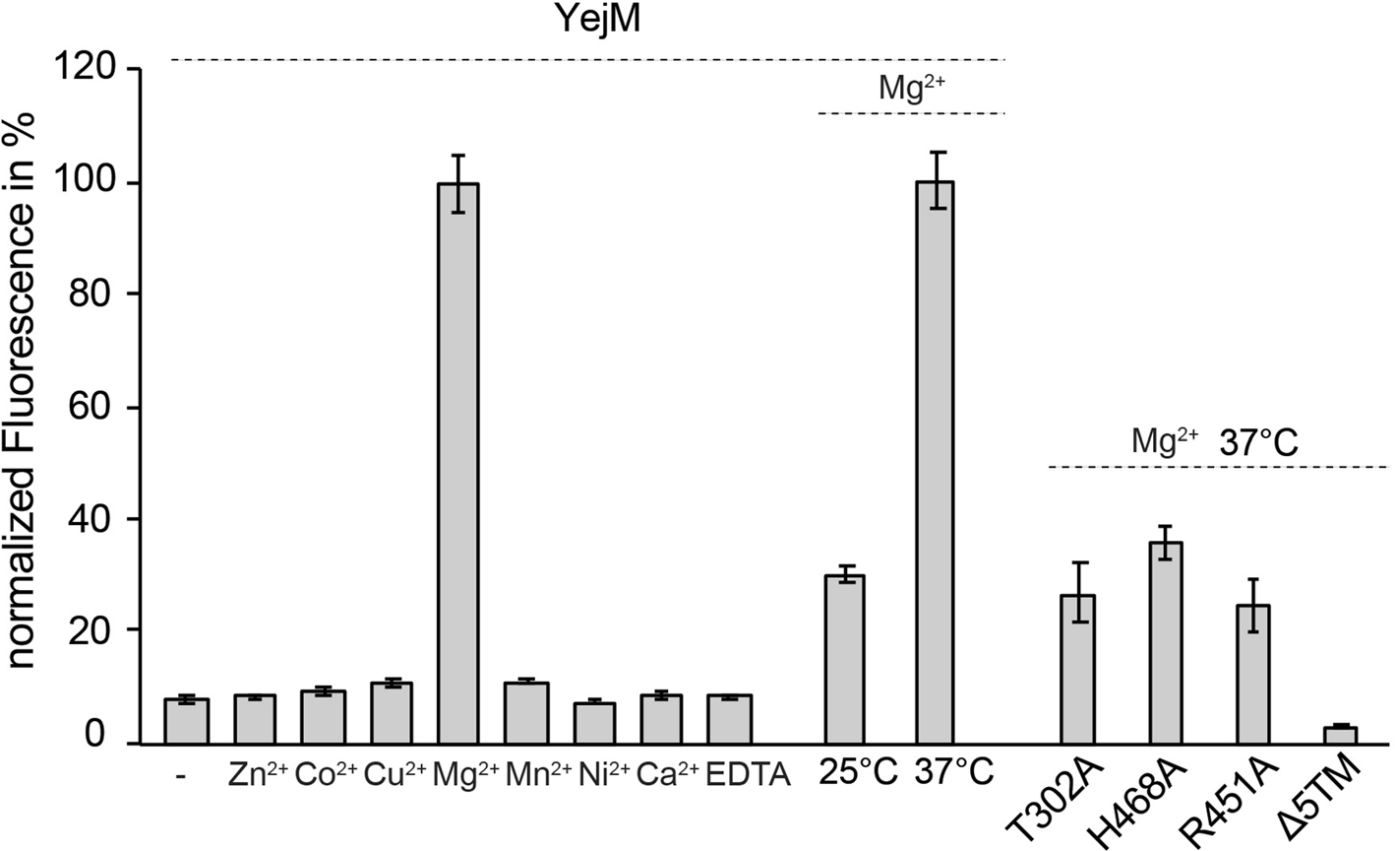
YejM is magnesium dependent phosphatase. Phosphatase activity of YejM in the absence and presence of various divalent metals and EDTA. Equimolar amounts of divalent cations (Zn^2+^, Co^2+^, Ni^2+^, Mn^2+^, Cu^2+^, Mg^2+^, and Ca^2+^), and EDTA were added to the reaction. The maximum activity (in the presence of Mg^2+^) was set to 100% and all other activities were scaled accordingly. Phosphatase activity of YejM at different temperatures (25 °C and 37 °C). Activity of YejM at 37 °C was set to 100% and activity at 25 °C was scaled accordingly. Phosphatase activity of wild type YejM, YejMT302A, YejMH468A and YejMΔ5TM. Activity of wild type YejM was set to 100% and remaining activities were scaled accordingly. All data are an average of three experimental replicates. Error bars indicate the standard deviation of the data.

Based on conservation, location of the active site, presence of divalent metal ion, and the conserved threonine Thr302, we hypothesized that YejM may have a phosphatase activity. To test whether YejM can hydrolyze phosphate groups, we chose the fluorogenic compound 6,8-Difluoro-4-Methylumbelliferyl Phosphate (DiFMUP) as a substrate. To assess whether enzymatic activity is dependent on metal ions, we performed the assay with and without equimolar amounts of salts of various divalent cations. Our data revealed for the first time that YejM has a phosphatase activity, which is highly specific to the presence of magnesium ions (Fig. 3). YejM in presence of magnesium ions (Mg^2+^) resulted in a 100-fold increase in the phosphatase activity compared to other metals or without additional metal ions or added EDTA (Fig. 3). Thus, YejM has a phosphatase activity that is specifically dependent on the presence of Mg^2+^.

The observed enzymatic activity of YejM is lower compared to potato acid phosphatase (PAP) (Supplemental Fig. 3), which may be attributed to the following two factors: i) DiFMUP might differ substantially from the natural substrate for YejM; ii) the absence of a second substrate serving as a phosphate acceptor molecule might slow down the substrate turnover rate of YejM. We tested whether the activity of YejM is temperature dependent and conducted the assay at 25 °C and 37 °C. Indeed, YejM has a 3.3-fold higher phosphatase activity at 37 °C as compared to the activity at 25 °C (Fig. 3). Higher activity at 37 °C suggests that YejM increases activity upon host-infection, at which point Gram-negative bacteria are exposed to body temperatures higher than 36 °C, and therefore YejM is most effective in OM modification for temperature adaptation and change in permeability.

We performed alanine substitution mutagenesis at active site residues and tested activity. Mutation of the conserved Thr302 to alanine resulted in about 70% loss of phosphatase activity (Fig. 3). Mutation of Arg451 to alanine, which is structurally located at a position similar to His453 and His476 in EptA and LtaS, respectively (Fig. 2b,e,f), resulted in an approximately 60% decrease of phosphatase activity (Fig. 3). A similar decrease in activity was observed upon substitution of His468 to alanine, which is proximate to the active site and the bound metal ion (Figs. 2c, 3). Next, we tested whether the periplasmic domain alone has enzymatic activity. YejMΔ5TM, which lacks the 5TM domain, showed a loss of ~ 97% phosphatase activity that is lower than YejM with divalent cations other than magnesium (Fig. 3). Our results suggest that both the active site and the 5TM domain including the RR linker region are essential for the magnesium dependent enzymatic activity of YejM.

YejM has been associated with increased amounts of cardiolipin in the OM and proposed to be part of an essential cardiolipin transport system^22, 32^. More recent studies have attributed YejM function linked to phoPQ-dependent role in lipopolysaccharide assembly^25^, LpxC homeostasis that ultimately regulates the balance of PL and LPS in the outer membrane^26–28^. Since we found an intact active site, bound metal ions and enzymatic activity, we asked the question whether changes of OM lipid composition are directly coupled to the enzymatic activity of YejM. To test this, we analyzed the lipid composition of IM and OM of *E. coli* Top10 cells grown under various conditions, and quantified the three major lipid components PE, PG and CL using thin layer chromatography (TLC) (Supplemental Figs. 3 and 4). Our results allowed us to conclude that YejM is likely not directly involved in specifically cardiolipin or other phospholipid translocation to the OM. We therefore suggest that YejM is probably not a cardiolipin transporter and its enzymatic activity is linked to its roles in the LPS and possibly other yet unknown pathways.

**Figure 4:**
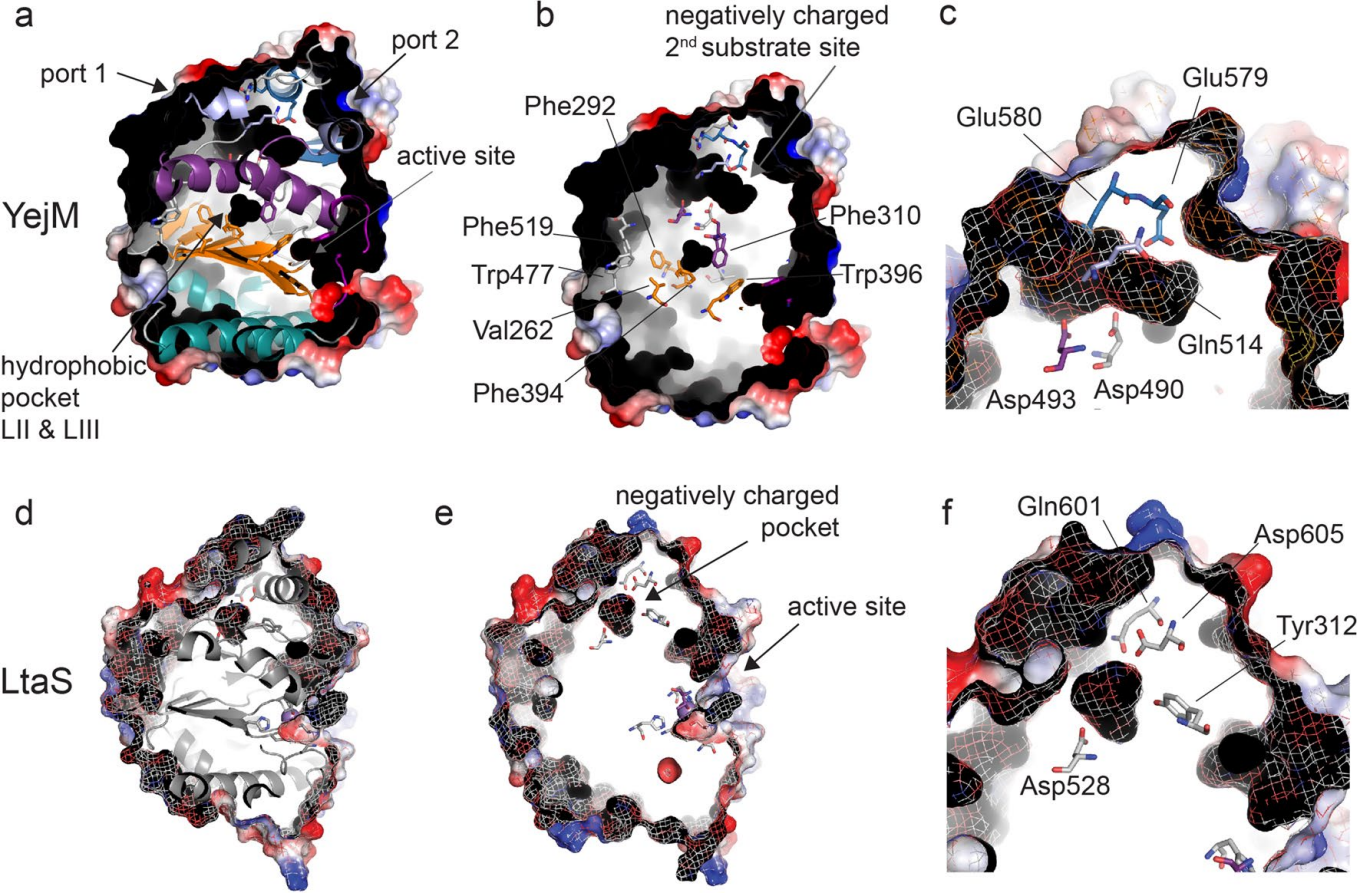
Secondary substrate pocket site of YejM in comparison to LtaS. (**a**) YejMPD viewed from the top showing cartoon representation of the peptide chain and electrostatic surface representation, structure is cropped in Z plane to show hydrophobic pocket (black arrow) and negatively charged secondary substrate side pocket and ports 1 and 2 (black arrows). Location of the active site is indicated by a grey arrow to the front of the PD. (**b**) YejMPD viewed from the top showing only electrostatic surface representation structure is cropped in Z plane to show hydrophobic pocket created by hydrophobic residues and negatively charged secondary substrate side pocket lined by negative charged residues (grey arrow). Location of the active site is indicated by black arrow to the front of the PD. Residues located around the negatively charged pocket and towards the active site are shown in stick representation. (**c**) Zoom in to the location of the negatively charged secondary substrate side pocket with residues in stick representation. (**d**) LtaS viewed from the top showing cartoon representation of the peptide chain and electrostatic surface representation in an overlay of solid and mesh surface, structure is cropped in Z plane to show a pocket at a similar location to YejMPD negatively charged pocket at proposed secondary substrate site. (**e**) LtaS in same the view as in D without cartoon representation of the main peptide chain. Residues lining a negatively charged pocket and the active site are shown in stick representation. (**f**) Zoom in to the location of the negatively charged pocket of LtaS with lining residues in stick representation.

### YejM has a potential secondary substrate site between the hydrolase and C-terminal domains

**Figure 5:**
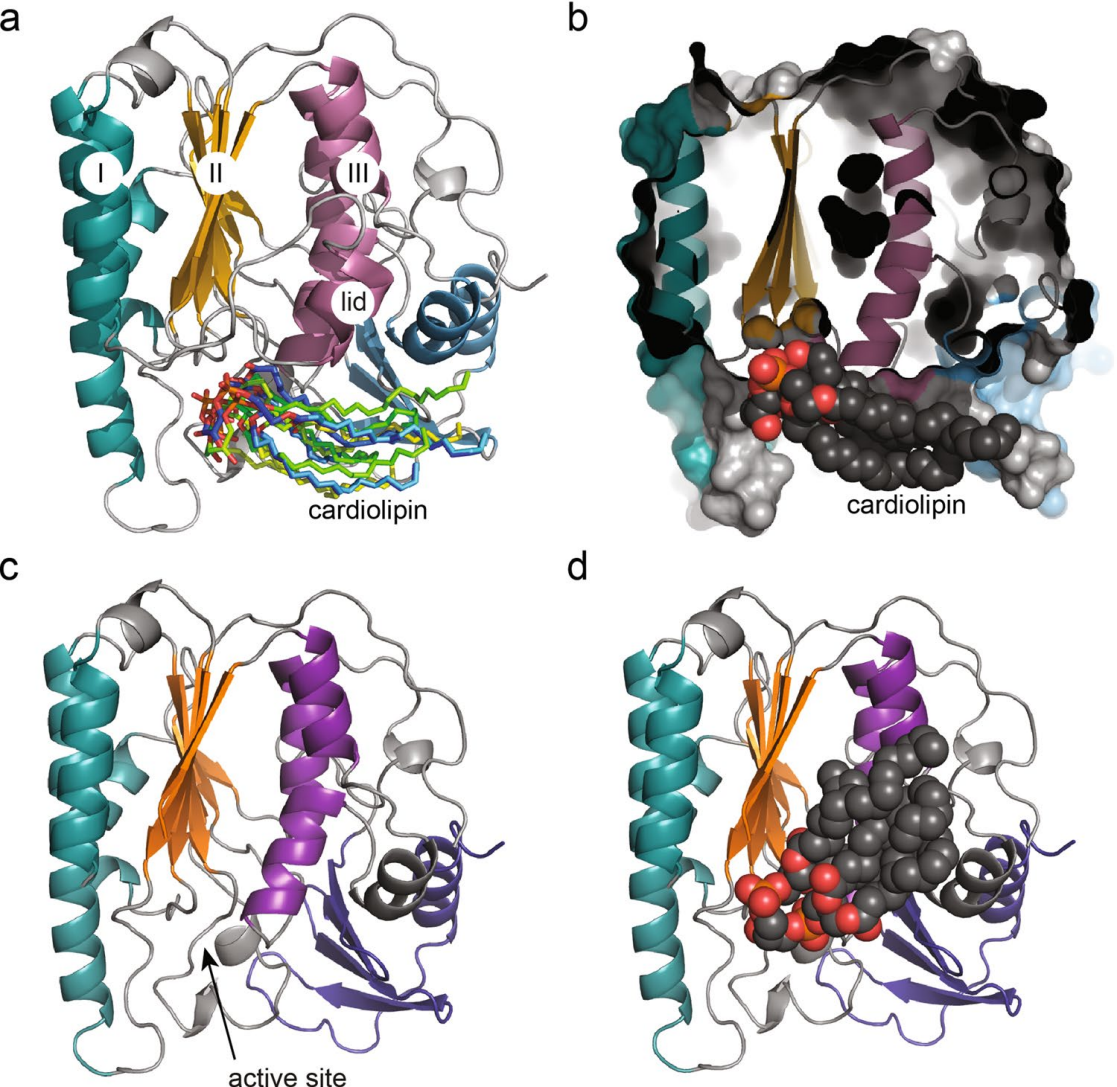
Cardiolipin docking to YejMPD with and without lid. (**a**) YejMPD structure indicating layer I, II and III, intact lid, and superimposed cardiolipin molecules in their preferred docked location and orientations, with phospholipid heads groups pointing towards the active site and acyl chains towards the CT domain. (**b**) YejMPD shown in cartoon and surface volume representation cropped in the Z-plane to visualize the vestibule of the active site, Black arrow point towards preferred location of cardiolipin molecule. Gray arrow points towards the hydrophobic pocket and proposed cardiolipin binding site between layers II and III. (**c**) YejMPD with removed lid, black arrow pointing towards the exposed area of the hydrophobic pocket between layers II and III. (**d**) YejMPD with removed lid shows cardiolipin molecule with preferred acyl chains flipped upwards towards the hydrophobic pocket while the phospholipid headgroups remain located towards the active site vestibule at the base of layers II and III.

To dephosphorylate Thr302 YejM might require binding of a second substrate or an accessory protein. Following the hypothesis that a secondary substrate or protein may bind to the PD, we analyzed existing cavities in our structures (Figs. 2d–l, 4, 5). A hydrophobic cavity located between layers II and III within the α/β hydrolase domain was proposed to bind the acyl chains of cardiolipin during translocation across the periplasmic space (Figs. 4, 5)^32^. This proposed cardiolipin binding pocket is lined by hydrophobic amino acids, namely, Val262, Phe292, Leu343, Phe394, Trp396, Val471, Leu473, Trp477, occurring on the β-sheets of layer II, and residues Leu309, Phe310, Leu334, Phe349, Tyr354, and Phe519 of layer III. Phe292 is essential for cell survival, and Phe275, Phe349, Phe362, and Trp396 are essential for growth in antibiotic-containing media^32^. A proposed lid including residues 345–370 is hypothesized to act as a gate that closes or opens for acyl chain binding into this hydrophobic cavity^32^ (Fig. 5). The position of this loop in the six monomers in the asymmetric unit of our YejMPD structures differ by as much as ~ 8 Å, similar to the previously reported structures^32^ (Supplemental Movie 1). In addition to the hydrophobic cardiolipin binding cavity, we observed an unidentified electron density located in a negatively charged cavity at the interface between the hydrolase and the CT domain in the YejMPDF349A structure (Fig. 2g–j). Contributing to its negatively charged electrostatic surface are residues Asp488, Asp490, Asp493, Gln514, Glu579, and Glu580 (Fig. 2k). This electron density remained unidentified despite our efforts using various methods including modeling and refining the structure with various possible ligands and identification by mass spectrometry. Interestingly, this negatively charged pocket is accessible by two mostly positively charged funnels in the structure (Fig. 2l). The location, electrostatic nature and its accessibility allows us to suggest that this intriguing site could be a second substrate. We looked for similar negatively charged pockets at similar locations in other structures and identified one in LtaS (Fig. 4a–f). No function is reported for this site for LtaS or other sulfatases and phosphatases. Our findings suggest that YejM may bind a second substrate that serves to dephosphorylate Thr302.

## Discussion

We have unveiled a new functional identity of the essential inner membrane protein YejM that is associated with changing the OM during host infection. The structure determination from our YejMPD crystals revealed a metal ion-containing active site that is located at the base of the hydrolase domain. Phylogenetic analysis of the PD sequences revealed that the active site is conserved across many members of the larger phosphatase super-family. Key active site residues were identified in YejM that serve as important catalytic residues and metal binder. Phylogenetic analysis and structure-based sequence determination around the active site revealed that the active site residues of YejM are highly conserved across homologues but also indicate changes that likely accommodate for different substrate and metal binding. Our enzymatic assay shows that YejM has magnesium dependent phosphatase activity. The integrity of the active site and the 5TM domain including the RR linker region are essential for YejM enzymatic activity. A second substrate binding site indicates that YejM might function in a multi-step process that combines enzymatic activity with OM remodeling pathways. Taken together, we assign a new functional identity to YejM: a metalloenzyme whose activity may be coupled to OM modifications. Our results raise intriguing questions about how YejM functions as an enzyme, the nature of its substrates and how it works on a molecular level, and how it is coupled to changes to the OM lipid environment. Our results fundamentally change how we think about this essential membrane protein that ultimately is granting survival of Gram-negative bacteria during infection.

Our extensive structure similarity search provided us with many enzymes to which we compared YejM with (Supplemental Tables 2 and 3). Despite medium to low overall sequence identity, the high sequence conservation at key active site residues allowed transfer of knowledge from better studied enzymes to begin to understand the newly identified enzymatic nature of YejM. YejM has previously been compared to an arylsulfatase and the lipoteichoic acid synthase LtaS^32^. Even with their striking structural similarities, the authors concluded that because of lack of crucial cysteine and serine residues and the lack of metal ions in their structures, YejM cannot function as an arylsulfatase and is likely not a metalloenzyme^32^. However, our structures, phylogenetic and biochemical data suggest that YejM is indeed a metalloenzyme with a conserved active site similar to LtaS, EptA and MCR1, and has phosphatase activity that is dependent on magnesium ions and the integrity of the active site (Figs. 2b–f, 3). Interestingly, the sequence of the YejM periplasmic domain is more closely related to LtaS in Gram-positive bacteria than pEtN transferases EptA and MCR-1 of Gram-negative bacteria (Fig. 1b), suggesting YejM and LtaS may be distant orthologs. Members of the larger alkaline phosphatase family balance specificity and promiscuity in their evolution around the active site resulting in multidimensional activity transitions that may also hold true for the sub-family YejM belongs to^36, 42, 43^.

Our data show divalent cations bound in YejMPD structures and that in the presence of Mg^2+^, YejM can remove the phosphate group from DiFMUP (Fig. 3). We speculate that the Mg^2+^-specific enzymatic activity of YejM was missed in earlier studies^22, 32^. Previously determined structures of *E. coli and S. typhimurium* YejMPD (PbgA) have not shown cation binding at the active site of YejM^32^. YejM was compared to LtaS, a lipoteichoic acid synthase that relies on Mg^2+^ ions to function, and to the arylsulfatase from *Pseudomonas aeruginosa* (PDB ID 1HDH)^44^. However, no cation ions were observed in the structures^32^ and together with no assessed enzymatic activity concluded that PbgA/YejM is not an enzyme^32^, agreeing with a previous work^22^. Our extensive comparison of PbgA/YejM to other enzymes and phylogenetic comparison contrast the fair suggestion of those previous studies that YejM/PbgA unlikely contains a metal binding site or has enzymatic activity^22, 32^. However, our crystal structures of YejMPD favored metal bound states of YejM, likely serendipitously through crystallization and crystal packing. Therefore, our structural data along with extensive phylogenetic and functional analysis provide a strong evidence that YejM/PbgA can bind divalent metal ions at the conserved active site necessary for its enzymatic activity. Notably, our structures show a less tight coordinated Mg^2+^ ion that suggests that the metal binding site is possibly reversible, harboring functional relevance that may regulate functionally distinct properties of YejM^25–28^. Mg^2+^ binding sites are known to have coordination plasticity, especially at binding sites that contribute mostly monodentate coordination^45^. This suggests that the YejMPD active site is likely subject to a changing interface that is modulated by the RR linker region and 5TM domain. It is possible that the YejMPD and RR linker regions undergo drastic conformational changes similar to what has been reported for EptA^12^.

**Figure 6:**
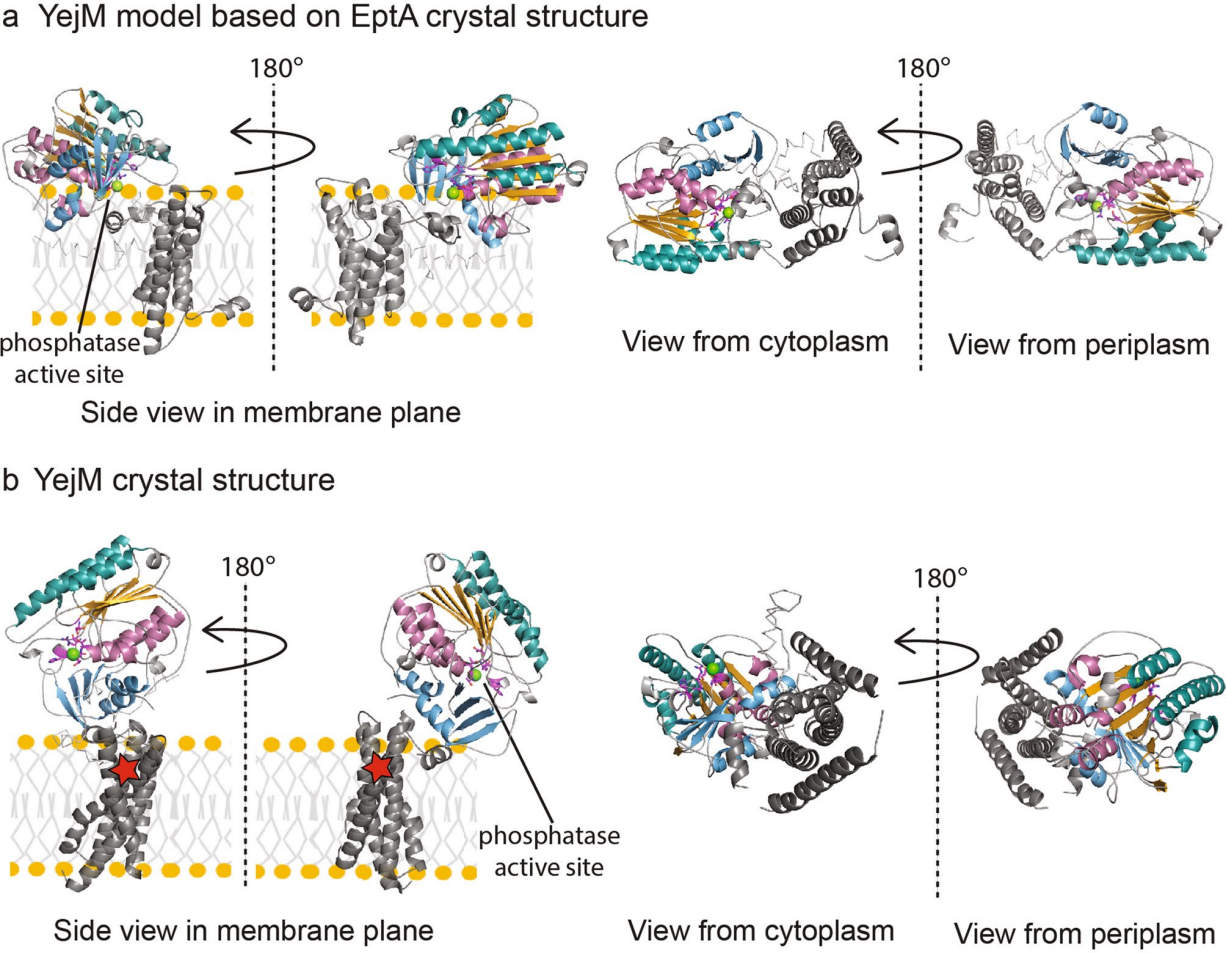
The periplasmic domain can likely adopt different orientations relative to the membrane. (**a**) From left to right, side views within the membrane plane rotated by 180° of YejM full-length model that is chimera between the model of the 5TM and RR linker domains based on the full-length crystal structure of EptA (PDB ID 5FGN) and the YejMPD from our original structure. Right, YejM model view on top of the membrane plane from the cytoplasmic side and rotated by 180° to view from the periplasmic side. Location of the active site is indicated by black line. (**b**) From left to right, side views within the membrane plane rotated by 180° of YejM full-length crystal structure (PDB ID 6V8Q). To show the active side discovered in our PD structure, we show only our PD that is superimposed on the PD of the YejM crystal structure (same as in a). The location of the suggested phospholipase site^34^ buried further in the TM5 portion is indicated by a red asterisk. Right, same composite YejM structure view on top of the membrane plane from the cytoplasmic side and rotated by 180° to view from the periplasmic side.

We hypothesize that the RR linker region between 5TM and PD plays a crucial role in creating a specific functional environment for substrate binding and function of YejM. Molecular dynamics studies of EptA revealed that the PD can exist in various orientations relative to the 5TM and membrane plane^12^. We created a chimera model that includes the 5TM and RR linker domains based on the full-length crystal structure of EptA (PDB ID 5FGN) and combined it with our YejMPD structure (Fig. 6a). This model visualizes the possible location of the active site relative to the 5TM domain and membrane plane (Fig. 6a). We then superimposed our YejMPD structure on the PD of the YejM crystal structure (PDB ID 6V8Q) to visualize the position and environment of the metal bound active site (Fig. 6b). The comparison of both suggests that the active site around Thr302 can be solvent exposed or buried towards the membrane (Fig. 6a,b and Supplemental Fig. 6).

Our data suggests YejM to be enzymatically closer to EptA and MCR-1 than LtaS. EptA and MCR-1 both hydrolyze the phosphoethanolamine (pEtN) from phosphatidylethanolamine (PE), to then transfer the pEtN group to lipid A, which neutralizes lipid A and renders binding of polymyxin-group antibiotics ineffective. EptA transfers pEtN to lipid A that is presented by another protein and may bind two differently sized substrates at its active site^12^. MCR-1/2 however are suggested to have a secondary substrate binding site and pEtN transfer to lipid A is facilitated by MCR proteins alone^33, 41, 46^. The latter may be the nature of the negatively charged cavity in YejM (Fig. 2g–l). We hypothesize that this negatively charged cavity serves as a secondary binding site for a substrate that acts as phosphate receptor to dephosphorylate Thr302. Interestingly, in our comprehensive in silico docking study and analysis based on the study by Planas-Iglesias^47^, most negatively charged phospholipid head groups of cardiolipin localize towards the active site (Fig. 5a–d). The acyl chain location flips towards the hydrophobic pocket between layers II and III when using a YejMPD model with missing lid sequence (Fig. 5c,d). However, we question whether the hydrophobic pocket can enlarge enough to accommodate four acyl chains of CL.

We observed that both YejM and YejMΔ5TM undergo proteolytic processing within the RR linker region. Such proteolytic cleavage can possibly inactivate the protein, as in the case of LtaS^48^, or change its function as observed in the case of phosphoglycerol transferase OpgB^49^. While no proteolytic processing has been reported for EptA and MCR-1 proteins^12, 50^, we suggest that this process may be of importance in allowing the two domains of YejM (5TM and PD) to interact with different proteins and substrates independent of each other. It is important to note that while YejM might undergo proteolytic processing, the phosphatase activity depends on the integrity of the protein. Notably, the PD alone does not have enzymatic activity (Fig. 3). This is in stark contrast to EptA, and LtaS, for which the periplasmic/extracellular domains alone were shown to retain phosphatase activity^51, 52^. Strikingly, although the soluble domains of both EptA and LtaS are able to hydrolyze their substrates, EptA cannot add phosphoethanolamine to lipid A^12^, and LtaS cannot synthesize lipoteichoic acid from glycerol phosphate monomers^48^ in the absence of their transmembrane domains.

Our study provides first important data giving a new functional identity to YejM. This study extends our understanding and provides a strong foundation for future structure-guided interdisciplinary approaches to find inhibitors specific to YejM, and consequently contribute to fight infectious diseases.

## Material and methods

### Mutagenesis

Site-directed mutagenesis were carried out to generate following mutants: YejMPD-F349A using forward (FW) and reverse (REV) primers: FW CTGTTTTCTTCGGATGGCGCCGCCAGCCCGCTTTATCGTC, REV GACGATAAAGCGGGCTGGCGGCGCCATCCGAAGAAAACAG; YejM-T302A using FW CATATGAGTTCAGGGAATACCGCTGATAACGGTATTTTCGGC, REV GCCGAAAATACCGTTATCAGCGGTATTCCCTGAACTCATATG; YejM-R451A using FW GTCGTGATCATTACCGCAGGAGCCGGCATACCGTTGACGCCG, REV CGGCGTCAACGGTATGCCGGCTCCTGCGGTAATGATCACGAC; and YejM-H468A using FW GTCGCAAGGTGCTCTGCAAGTAC and REV CAGTCGAAGCGATTTTCTTC. The mutagenesis sites are underlined. PfuTurbo DNA polymerase (Agilent Technologies) and Q5 Hotstart Mutatagenesis kit (NEB) were used.

### Expression and purification of YejM constructs

Full-length YejM cloned in pBAD24 vector and its mutants were expressed in *E. coli* Top10 cells and purified as described in^22^. YejMPD and its mutants, cloned in pET28 vector, were expressed in *E. coli* BL21(DE3) cells and purified as described in^53^.

### Crystallization, X-ray diffraction data collection and processing

Purified YejMPD wild type and mutant proteins were crystallized according to^53^. The final crystallization conditions were derivatives of condition C6 of PEG-Rx HT (Hampton Research, catalog no. HR2-086) which consisted of 0.1 M HEPES pH 7.5, 12% w/v Polyethylene glycol 3,350. YejMPD crystal used for data collection appeared in a condition consisting of 0.1 M HEPES pH 6.8, 13% PEG 3350; whereas YejMPD-F349A crystals were obtained from the condition consisting of 0.1 M HEPES pH 6.8, 5% PEG3350. Crystals were harvested using Litho loops (Molecular Dimensions) or Nylon loops (Hampton Research), and frozen in liquid nitrogen without or with paraffin as cryo-protectant. Diffraction data were collected at the Advanced Light Source beamline 4.2.2 in Berkeley, CA at 100 K, using an oscillation of 0.1°–0.2° per image. Diffraction images were indexed and integrated using XDS^54^ and scaled and merged with Aimless using the CCP4 program suite^55^.

### Structure determination

The structure of YejM241-586 from data set YejMPD was solved in space group *P* 3_2_21 by molecular replacement using Phaser^56^ with a monomer from the crystal structure of *Salmonella* PbgA globular domain 191 (PDB code 5I5F^32^) as a search model. The structure was further refined using phenix.refine^57^. Non-crystallographic symmetry (NCS) restraints were imposed in the early stages of refinement and released and instead Translation-liberation-screw motion (TLS) were applied during later stages of refinement. The structure was refined to a resolution of 2.35 Å. Structure of YejMPD-F349A was determined by molecular replacement using a monomer of YejMPD (PDB ID 6VAT) as a search model. The structure of YejMPD-F349A mutant was refined to 2.05 Å. The refinement statistics for both structures are given in Table 1. Structures were deposited to the PDB database (https://www.rcsb.org/) and the following PDB IDs were assigned: 6VAT, and 6VC7.

### Phylogenetic analysis

The starting point of the phylogenetic analysis of YejM-related proteins were the *Salmonella typhimurium* cardiolipin transport protein YejM (aka PbgA) sequence segments of interest:

 > Se_YejMmotifDG = Se_YejM 261–288, DG at 268–269

NVLLITVDGLNYSRFEKQMPELATFAEQ

 > Se_YejMmotifTD = Se_YejM 290–311, TD at 302–303

IDFTRHMSSGNTTDNGIFGLFY

 > Se_YejMmotifGR = Se_YejM 443–471, GR at 450–451

TVVIITAGRGIPLTPEENRFDWSQGHLQV

 > Se_YejMmotifCTr = Se_YejM residues 526–586 (C-terminal domain)

NWVTAADGSTLAITTPQMTLVLNNNGHYQTYDLHGEKIKDQKPQLSLLLQVLTEEKRFIAN

The first three segments contain conserved active site residues DG, TD, and GR motifs, respectively, while the last segments constitute the C-terminal domain of unknown function. Each of these sequences were used as input to *jackhmmer* of the HMMER v3.2.1 package (www.hmmer.org) in a search against the UniProt TrEMBL database (release 2019_04; locally installed) to derive profile hidden Markov models. With default parameters, a total of 2,933 sequences were identified in TrEMBL with significant matches to at least one of the motifs.

The alignment output of *jackhmmer* was processed to identify unique sequences. For example, there were 66 distinct sequences matching the DG motif and occurring at least four times. The input Se_YejMmotifDG occurred in 165 of the 2933 TrEMBL sequences. These numbers reflect both redundancy of sequencing efforts (e.g., multiple recoveries of the entire YejM sequence from different samples) as well as evolutionary conservation across distinct organisms.

### Enzymatic assays

A 10 mM stock solution for DiFMUP substrate was prepared in N,N-dimethylformamide (DMF), stored at − 20 °C, and used within 1 month. This stock was diluted to 200 μM to 1 mM in the respective reaction buffer just before use. For measuring the activity of YejM, YejM-T302A, YejM-R451A, and YejM-H468A, the reaction buffer consisted of 25 mM tris, pH 7.5, 150 mM NaCl, and 0.015% DDM. For the periplasmic domain YejMΔ5TM, the reaction buffer used was 50 mM tris, pH 8.0, and 150 mM NaCl. In a typical experiment, 20 μM protein was incubated with 100 μM DiFMUP for a minimum of four hours in the dark. The temperature of incubation varied between 25 and 37 °C according to the experiment. Metal ion screen were conducted in the presence of equimolar amounts of salts of various divalent cations (ZnCl_2_, CoCl_2_, NiSO_4_, MnCl_2_, CuCl_2_, MgCl_2_, and CaCl_2_), or EDTA. Once the importance of Mg^2+^ ions in the phosphatase activity of YejM was confirmed, MgCl_2_ was included in 20X molar excess as compared to the protein in all subsequent assays. Appropriate negative controls (reaction buffers without protein and with the same amount of MgCl_2_ and DiFMUP) were used for each experiment. Potato acid phosphatase (PAP) included in the assay kit was used as a positive control, at a working concentration of 0.1–1 U/ml. The reaction buffer for PAP was 50 mM sodium acetate, pH 5.0. All reactions were set in 100 μL volume, in 96-well black, flat bottom microplates (Greiner Bio-one, cat. no. 655209) covered with lids. The fluorescence of the product of the enzymatic reaction, 6,8-difluoro-4-methylumbelliferone (DiFMU), was measured using Synergy Neo2 plate reader (BioTek) by excitation at 358 nm and emission at 455 nm.

All experiments were conducted in triplicates and the average values were reported. Results from different experiments were compared by setting the final fluorescence value of the sample with maximum fluorescence to 100% and adjusting fluorescence values of other samples accordingly.

### In silico binding studies

The ideal coordinates of cardiolipin (CDL) were downloaded from the HIC-Up server^58^ as a PDB file. AutoDock Tools (ADT) (version 1.5.6)^59^ was used to add polar hydrogens to the protein and ligand molecules, calculate Gasteiger charges, and finally generating PDBQT files. ADT was also used to determine the dimensions of a grid box encompassing the entire protein chain. Actual docking was done using AutoDock Vina^60^ using default docking parameters. AutoDock Vina generates the output of docked ligand (9 conformations) in a PDBQT file, which was later converted in a PDB file and visualized using UCSF Chimera^61^ and PyMOL (The PyMOL Molecular Graphics System, Version 2.0 Schrödinger, LLC). The contacts between receptor and ligand molecules in various docked poses were determined using Contact program in CCP4 suite^55^.

## Supplementary information

Supplementary file 1.

Supplementary file 2.

Supplementary file 3.

## Data Availability

Atomic coordinates and structure factors are deposited at the Protein Data Bank under the access codes 6VAT, 6VC7, 6VDF. Clones used in this study are available upon request to the corresponding authors.
