# Supplementary material for: The essential inner membrane protein YejM is a metalloenzyme
Source: Sci Rep. 2020 Oct 20;10:17794. doi: 10.1038/s41598-020-73660-6 (PMC7576196; doi:10.1038/s41598-020-73660-6)
Supplement: Supplementary file 1 [file 41598_2020_73660_MOESM1_ESM.pdf]

Supplemental material

Title: "The essential inner membrane protein YejM is a metalloenzyme"

Authors: Uma Gabale[1**], Perla A. Peña Palomino[1], HyunAh Kim[1], Wenya Chen[1], Susanne Ressl[1,2*]

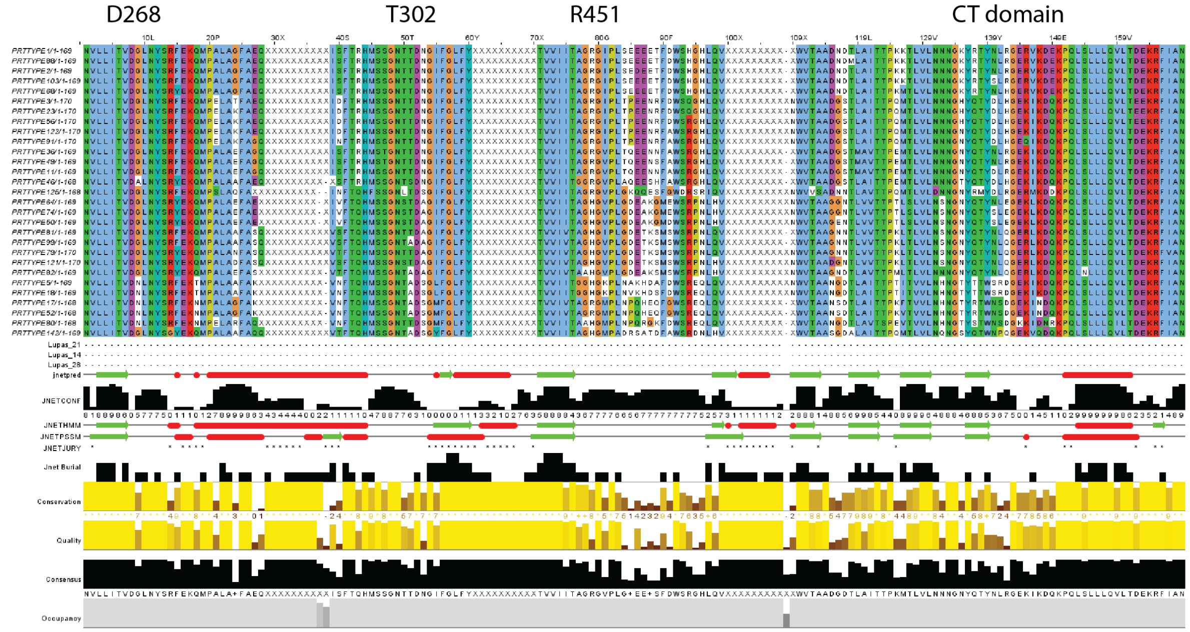

Supplemental Figure 1 Sequences around the active site residues and CT-domain, their conservation and natural occurring variations. The first stretch of sequence is centered around aspartate D268, the second around threonine T302, the third around arginine R451, and the fourth covers the CT domain.

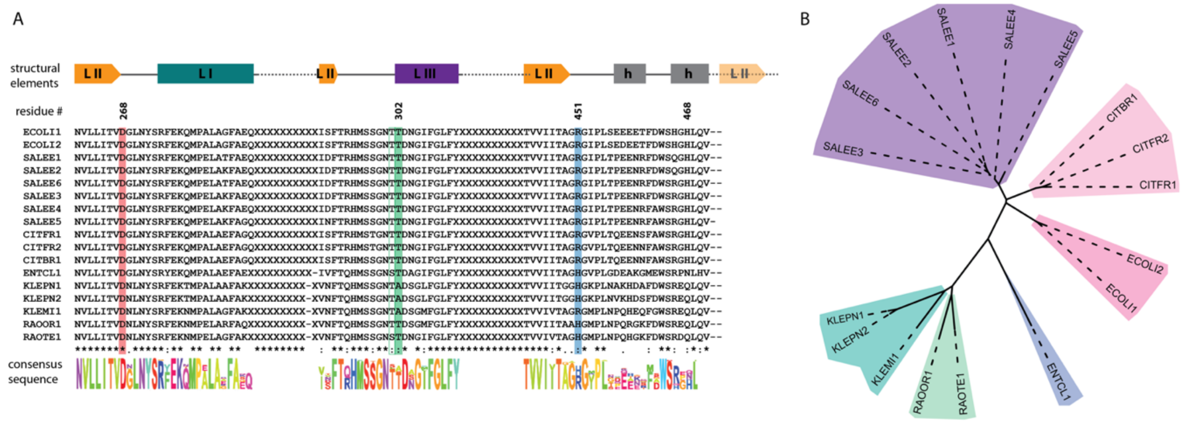

Supplemental Figure 2 Active site residue conversation across YejM homologues. A Sequence alignment of all three sequences around residue Asp268 (underlaid with red box), Thr302 (underlaid with green box), Arg451 (underlaid with blue box). Structural elements that guided our choice of sequence boundaries around those sites are indicated on the top representing beta-sheets with right facing rectangular arrow, helices with rectangles, and loops elements with a single line. Spaces between the sequence elements are marked with a dotted line. The color of the secondary elements reflects the same color code for layers I, II and III as in Figure 1. Layer I turquoise, layer II orange and layer III deep purple. The consensus sequence is shown at the bottom of the sequence, the color scheme is random and for better readability only. **B** Phylogenetic tree of YejM homologues. The length of branch lengths reflects distances between the clades. The unrooted tree groups YejM homologues of *Klebsiella pneumoniae* (KLEPN), *Klebsiella michiganensis* (KLEMI), *Raoultella terrigena* (RAOTE) and *Raoultella ornithinolytica* (RAOOR) into one clade (blue-green), *Enterobacter cloacae* (ENTCL) builds a separate outgroup (blue), various strains of *Salmonella* (SALEE1-5) together with *Escherichia coli* (ECOLI) and *Citrobacter braakii* and *freundii* (CITBR, CITFR, respectively), build a large clade (pink to purple). Phylogenetic tree was created with iTol (itol.embl.de) [1].

.

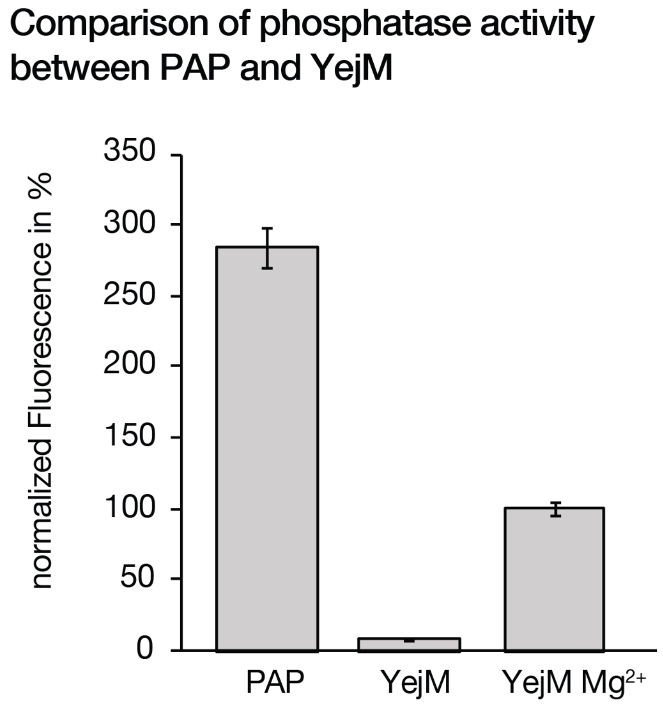

Supplemental Figure 3 YejM enzymatic activity for substrate DiFMUP in comparison to potato acid phosphatase (PAP). YejM enzymatic activity is presented in normalized fluorescence units (y axis). YejM with additional Mg^{2+} has a 1/3 o*f* PAP enzymatic activity for this substrate and YejM without addition of Mg^{2+} has close to no observable activity.

Supplemental Figure 4

a

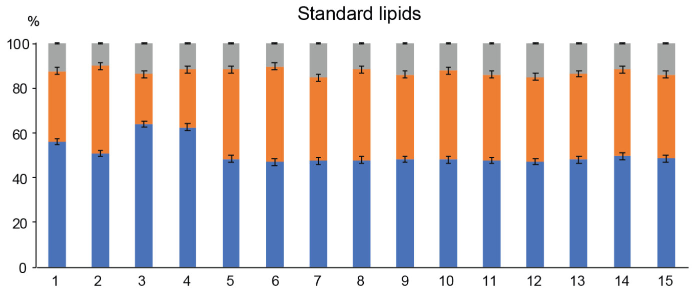

b

TLC #	TLC name	% PE	% PG	% CL
1	20200623_D268A_T302A	56.05	31.55	12.41
2	20200623_D268A+Mg_T302A+Mg	50.89	39.07	10.04
3	20200623_pBAD24_pBAD24+Mg	63.97	22.31	13.71
4	20200623_Top10_Top10+Mg	62.44	25.87	11.69
5	20200624_D268A+Ca_T302A+Ca	48.38	40.04	11.58
6	20200624_Top10+Ca_pBAD24+Ca	47.06	42.77	10.17
7	20200625_R451A_R451A+Mg	47.58	37.04	15.38
8	20200625_wt_wt+Mg	47.92	40.48	11.60
9	20200626_H468A+Ca_CTD+Ca	48.20	37.84	13.96
10	20200626_wt+Ca_R451+Ca	48.19	39.67	12.14
11	20200629_CTD_CTD+Mg	47.75	38.27	13.98
12	20200629_H468A_H468A+Mg	47.23	37.72	15.05
13	20200630_191_191+Mg	48.17	38.25	13.58
14	20200630_191+Ca_pET28a+Ca	49.66	38.63	11.71
15	20200630_pET28a_pET28a+Mg	48.54	37.55	13.90
	**STDEV**	**5.67**	**5.80**	**1.66**
	**STDERR**	**1.52**	**1.55**	**0.44**

% OM Lipids

c

LB medium with no supplement

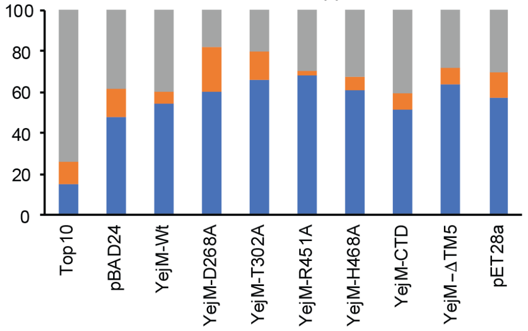

% IM Lipids

d

LB medium with no supplement

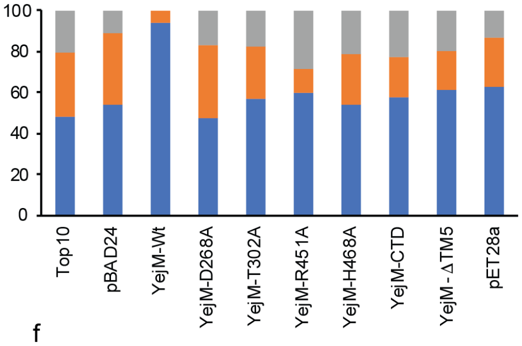

e

LB medium supplemented with 10 mM $MgCl_2$

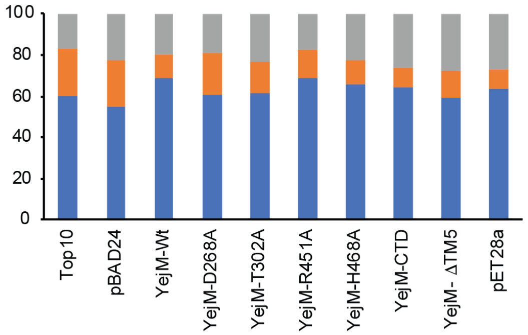

f

LB medium supplemented with 10 mM $MgCl_2$

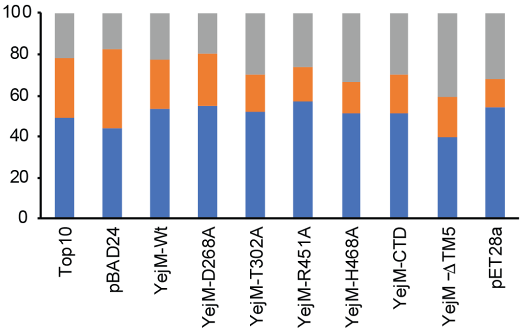

g

LB medium supplemented with 10 mM $CaCl_2$

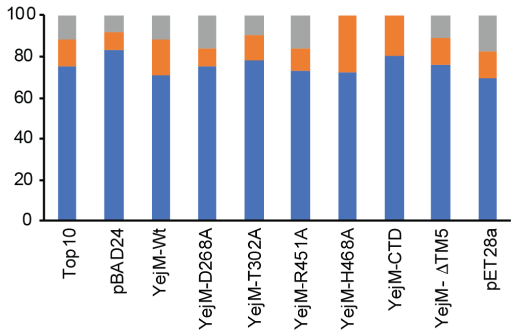

h

LB medium supplemented with 10 mM $CaCl_2$

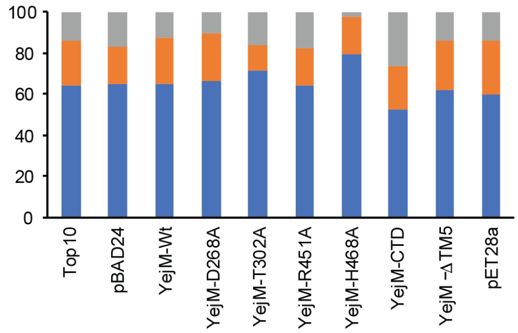

■ % PE ■ % PG ■ % CL

Supplemental Figure 4: a. Bar graph showing variation in the percentage intensities of standard phospholipids (PE, PG, CL) in all TLCs. Error bars are indicated on the graph. 20 µL of a mixture of three phospholipids (PE, PG, CL;1.67 mg/ml each) was spotted on TLC plates, developed and stained. The intensities of individual spots were calculated in ImageJ. b: percentage intensities of standard phospholipids and standard deviation and standard error of the data.
c-h. Bar graph showing variation in the percentage intensities of three major phospholipids. c. OM phospholipids from cells grown in LB medium without any supplement. d. IM phospholipids from cells grown in LB medium without any supplement. e. OM phospholipids from cells grown in LB medium supplemented with 10 mM $MgCl_2$. f. OM phospholipids from cells grown in LB medium supplemented with 10 mM $MgCl_2$. g. OM phospholipids from cells grown in LB medium supplemented with 10 mM $CaCl_2$. h. OM phospholipids from cells grown in LB medium supplemented with 10 mM $CaCl_2$.

Supplemental Figure 5 TLC plates

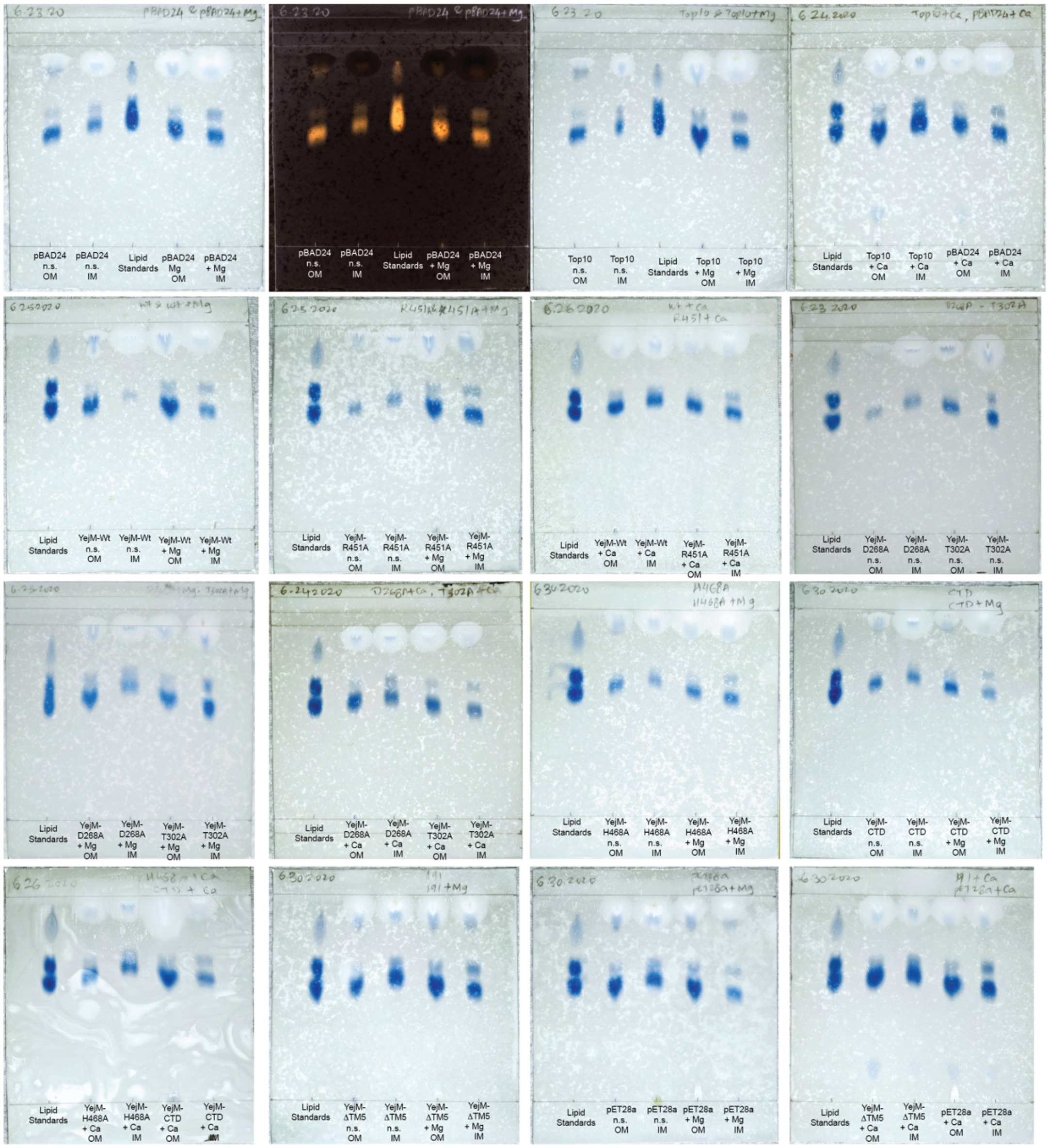

Supplemental Figure 5 TLC plates. Original images of TLC plates stained with molybdenum blue. Images were analyzed in ImageJ after inverting as shown in the example. Each lane represents one sample, indicated underneath. The membrane sample (OM/IM) and the culture conditions are indicated. n.s.: LB media with no supplement; Mg: LB media supplemented with 10 mM $MgCl_2$; Ca: LB media supplemented with 10 mM $CaCl_2$.

Supplemental Figure 6

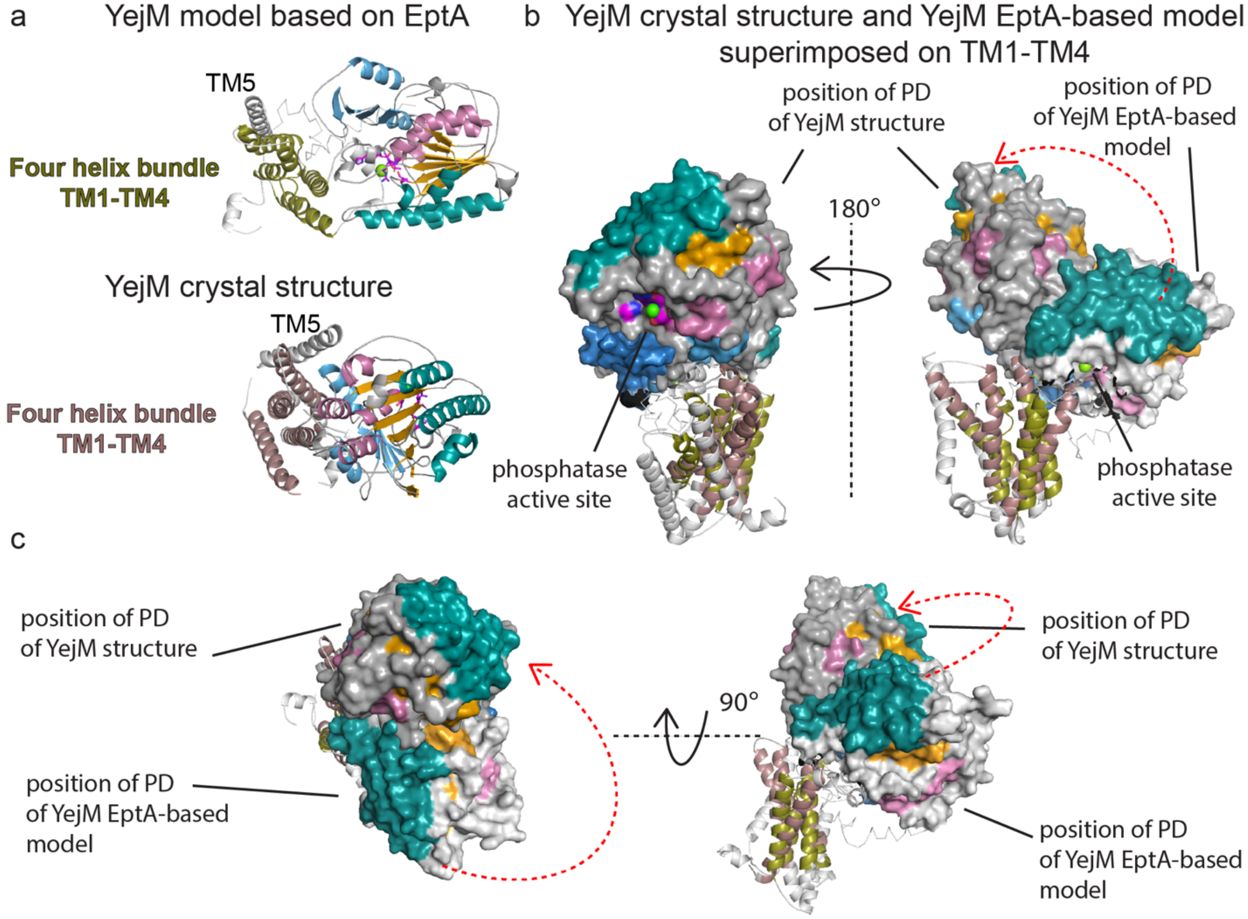

Supplemental Figure 6 Superimposition of YejM EptA-based model (EptA-YejM) with YejM crystal structure (PDB 6V8Q). **a** EptA-YejM and YejM crystal structure shown viewed from the periplasm. The four helix bundle within the transmembrane domain used for superimposition of both structures are indicated in yellow (EptA-YejM) and grey-magenta (YejM). TM5 (grey) did not superimpose well in the overall alignment and therefore was not taken into account for this superimposition that aims to visualize the different position of the PD in both structures (shown in surface representation). **b** EptA-YejM and YejM structures that were superimposed on TM1-TM4 in side view and rotated by 180°. To visualize the phosphatase active site with its bound putative Mg^{2+} only our PD structures are visualized. Left side with the PD active site exposing the putative Mg^{2+} to the solvent is our PD structure superimposed on the YejM PD of PDB ID 6V8Q, on the right side shows the view of the more buried PD active site enclosing the putative Mg^{2+} more towards the membrane and transmembrane domain. A dashed red arrow shows the difference in location between PD domain location in the EptA-YejM model and YejM crystal structure. **c** Superimposed structures viewed from the periplasm (right) and rotated by 90° into side view (left). Black lines indicate location of PD of either the EptA-YejM or YejM crystal structures. Red dash lined arrows indicate the difference in location of the PD.

Supplemental Table 1: X-ray data collection and refinement statistics for YejMPD twinned.

Important note from the authors: No conclusions were drawn from this structure in this manuscript. This structure may be of interest to the community due to its bound metals and substrate molecules. Twinned crystal and low data redundancy lead to a less optimal data and model refinement statistics as expected for this resolution range. We therefore advise to interpret this structure with caution.

	**YejMPD-twinned**
PDB ID	6VDF
Data collection	ALS BEAMLINE 4.2.2
Wavelength (Å)	1.00003
Space group	P 1
**Cell dimensions**	
a, b, c (Å)	42.948, 42.957, 181.209
α, β, γ (°)	94.28 94.015 111.779
Resolution (Å)	59.9-1.92 (1.989-1.92)
R_{merge}	0.08983 (0.7619)
I / σI	7.85 (1.07)
CC1/2	0.972 (0.191)
Completeness (%)	77.15 (60.59)
Redundancy	1.6 (1.4)
**Refinement**	
Resolution (Å)	59.9 - 1.92 (1.989 - 1.92)
No. reflections	70265 (5488)
R_{work} / R_{free}	0.2141/ 0.2639
No. atoms	11745
Protein	10872
Ligand/ion	61
Water	812
B-factors	20.42
Protein	19.82
Ligand/ion	47.33
Water	26.37
R.m.s deviations	
Bond lengths (Å)	0.002
Bond angles (°)	0.48
**Ramachandran statistics**	
favored (%)	90.47
allowed (%)	7.84
outliers (%)	1.69

*Statistics for the highest-resolution shell are shown in parentheses.

Supplemental Table 2: DALI comparison of YejM PD to other hydrolases/isomerases/transferases

PDB	Protein name	Enzyme family	Organism	Z score	rmsd	lali	nres	%id	Metal ion
6j66	Chondroitin sulfate/dermatan sulfate endolytic 4-O-sulfatase	Hydrolase	*Vibrio sp.* FC509	30.7	2.7	308	476	17	Ca^{2+}
3b5q	putative sulfatase	Hydrolase	*Bacteroides thetaiotaomicron*	30.5	2.6	305	467	17	Zn^{2+}
4miv	Sulfamidase	Hydrolase	*Human*	30.3	2.8	305	480	18	Ca^{2+}
3lxq	Uncharacterized protein	--	*Vibrio parahaemolyticus* serotype O3:K6 (strain RIMD 2210633)	30.2	3.4	316	409	14	Cl^{-}
6hr5	S1_25 family sulfatase module of the rhamnosidase FA22250	Hydrolase	*Formosa agariphila*	29.9	2.9	304	376	16	Ca^{2+}
2w5q	LtaS	transferase	*Staphylococcus aureus*	29.7	3.3	304	424	17	Mn^{2+}
5g2v	N-ACETYL GLUCOSAMINE-6-SULFATASE	Hydrolase	*Bacteroides thetaiotaomicron*	29.1	2.7	306	494	17	Ca^{2+}
4upl	sulfatase SpAS2	Hydrolase	*Silicibacter pomeroyi*	28.2	2.7	307	555	14	Zn^{2+}
5fql	iduronate-2-sulfatase	Hydrolase	Human	28.1	2.9	302	507	19	Ca^{2+}
1p49	Placental Estrone/DHEA Sulfatase	Hydrolase	Human	27.6	3.1	304	549	15	Ca^{2+}
4cys	G6 mutant of PAS, arylsulfatase	Hydrolase	*Pseudomonas Aeruginosa*	24.4	3.5	309	534	17	Ca2+
5olt	extramembrane domain of the cellulose biosynthetic protein BcsG	Transferase	*Salmonella typhimurium*	22.1	3.5	275	383	14	Zn^{2+}
6c01	ectonucleotide pyrophosphatase / phosphodiesterase 3	Hydrolase	human	20.1	2.9	247	819	13	Zn^{2+}, Na^{+}, Ca^{2+}

5xwk	alkaline phosphatase	hydrolase	*Sphingomonas*	20.1	2.8	239	530	15	Zn^{2+}, Ca^{2+}
4b56	ectonucleotide pyrophosphatase-phosphodiesterase-1 (NPP1)	Hydrolase	*Mus musculus*	19.3	2.9	247	816	11	Zn^{2+}, Ca^{2+}
6dgm	phosphoglycerol transferase GacH	Transferase	*Streptococcus pyogenes*	18.5	4.0	266	382	12	Mn^{2+}, Ca^{2+}
3igy	phosphoglycerate mutase	Isomerase	*Leishmania mexicana*	17.5	3.1	228	549	16	Na^{+}, Co^{2+}
5kxa	Ectonucleotide pyrophosphatase/ phosphodiesterase	HYDROLASE/ HYDROLASE INHIBITOR	human	17.2	2.9	237	751	13	Zn^{2+}, Ca^{2+}
4n7t	phosphopentomutase	ISOMERASE	*Streptococcus mutans*	17.0	3.0	217	402	15	Mn^{2+}
5m0s	Ectonucleotide pyrophosphatase/phosphodiesterase	HYDROLASE	*Rattus norvegicus*	16.9	2.9	236	777	12	Zn^{2+}, Na^{+}, Ca^{2+}
2dlg	Acid Phosphatase A	Hydrolase	*Francisella tularensis*	15.8	3.1	221	481	15	Unknown
3e2d	alkaline phosphatase	Hydrolase	*Vibrio*	15.7	3.1	217	502	14	Zn^{2+}, Mg^{2+}
2zkt	phosphoglycerate mutase	ISOMERASE	*Pyrococcus horikoshii*	15.8	2.8	204	381	19	Zn^{2+}, Ca^{2+}

Supplemental Table 3: DALI comparison of C-terminus of YejM (501-586) to PDB database

PDB	Protein name	Z score	rmsd	lali	nres	%id
6j66	Chondroitin sulfate/dermatan sulfate endolytic 4-O-sulfatase	5.2	2.9	79	476	9
1a87	COLICIN N;	5.0	3.1	62	297	8
4o56	SERINE/THREONINE-PROTEIN KINASE PLK1;	4.9	6.3	64	244	11
5lhx	SERINE/THREONINE-PROTEIN KINASE PLK4;	4.8	2.9	60	87	5
2n19	SERINE/THREONINE-PROTEIN KINASE PLK4;	4.6	4.0	60	87	8
5jov	ALPHA-XYLOSIDASE BOGH31A;	4.5	5.7	53	949	15
4nkb	PROBABLE SERINE/THREONINE-PROTEIN KINASE ZYG-1;	3.9	3.4	60	204	2
5wlz	DNA REPAIR PROTEIN XRCC4, MYOSIN-7;	3.9	4.1	59	206	3
4cys	ARYLSULFATASE;	3.8	3.8	77	534	12
6hr5	ALPHA-L-RHAMNOSIDASE/SULFATASE (GH78);	3.8	3.0	81	376	5
4n7v	SERINE/THREONINE-PROTEIN KINASE PLK4;	3.7	2.5	58	222	5
4upl	SULFATASE FAMILY PROTEIN;	3.7	3.0	83	555	7
6dhx	TIPC2;	3.7	2.7	68	182	4
6qhg	POLYMERASE;	3.6	2.6	52	119	8
1p49	STERYL-SULFATASE;	3.6	3.5	82	549	7
5xq3	PCRGLX PROTEIN;	3.4	7.1	55	901	16
4bxr	CPAP;	3.4	3.4	53	183	6
2obd	CHOLESTERYL ESTER TRANSFER PROTEIN;	3.3	5.9	60	472	10
5wd6	SHORT PALATE, LUNG AND NASAL EPITHELIUM CARCINOMA	3.1	3.5	65	195	11
3cbt	PHOSPHATASE SC4828;	3.1	4.1	62	210	5
4qtq	XAC2610 PROTEIN;	3.1	3.5	57	209	12
5hv1	PHOSPHOENOLPYRUVATE SYNTHASE;	3.1	4.1	68	847	6
2x2h	ALPHA-1,4-GLUCAN LYASE ISOZYME 1;	3.0	5.0	47	1025	11
2j6h	GLUCOSAMINE-FRUCTOSE-6-PHOSPHATE AMINOTRANSFERASE	3.0	4.5	59	609	8
6j9f	DNA-DIRECTED RNA POLYMERASE SUBUNIT ALPHA;	3.0	8.3	62	1296	11
5mus	L PROTEIN;	3.0	6.9	59	328	12
3dcz	PUTATIVE RNFG SUBUNIT OF ELECTRON TRANSPORT COMPL	3.0	2.4	52	170	2
5y83	MEMBRANE PROTEIN INSERTASE YIDC;	3.0	2.1	43	342	12
1f34	PEPSIN A;	3.0	4.9	54	138	2

Supplemental Movies

Movie 1 file name: SMovie1.mov
Morph between chain C and D of YejM the structure with six monomers in the asymmetric unit. Both chains represent the most “closed” or “open” conformation of the lid.

Movie 2 file name: SMovie2.mov
Same morph as in movie 1, this movie highlights the movement of aromatic residues (red) located at layers II and III, and one aromatic residue (blue) located between layer I and II.

Supplemental Material and Methods

Crystallization and structure determination of YejMPD from twinned dataset

YejMPD-twinned crystals used for data collection appeared in a condition consisting of 0.1 M HEPES pH 7.0, 9 % PEG3350. The structure of YejMPD from twinned data set was solved in space group *P* 1 by molecular replacement in Phaser using a monomer of YejMPD (PDB ID 6VAT) as a search model, and refined to a resolution of 1.92 Å with applied detwinning operator *(-h,-k,h+k+l)* but otherwise following similar TLS refinement strategies as for YejMPD.

Membrane lipid isolation

Total membranes were isolated from 1 L cultures of different strains of *E. coli* as per the protocol described in[2]. Separation of inner and outer membrane fractions were performed as described by[3]. Each membrane pellet was resuspended in 3 ml ddH_2O and ultra-centrifuged at 50000 rpm for 45 min at 4 ^{O}C. After removing the supernatant, each membrane pellet was resuspended in 2 ml ddH_2O. To this, 200 μL of 5% sarkosyl was added and incubated for 20 min at room temperature with shaking. Ultracentrifugation at 50000 rpm for 45 min at 4 ^{O}C yielded the inner membrane fraction in the supernatant. The pellet, that represents the OM fraction, was washed with 2 ml of 1% sarkosyl and resuspended in 2 ml ddH_2O.

Lipids were isolated from the IM and OM fractions by the method of Bligh and Dyer[4]. Briefly, to each 1 ml of sample in a glass vial, 3.75 ml 1:2 (v/v) chloroform:methanol was added and mixed by vortexing. This single-phase Bligh-Dyer layer was converted into a double phase by adding 1.25 ml Chloroform followed by 1.25 ml ddH_2O and vortexing. The two layers were separated by centrifuging the glass vials at 2000 RPM in a table-top centrifuge for 10 min at room temperature. The bottom phase was recovered by carefully aspirating with a glass pasture pipette and transferring to another glass vial. The original aqueous phase was washed with another 1.25 ml of Chloroform and vortexing and centrifuging as before. The organic phase from this wash was mixed with the previous one. The lipids were dried under vacuum and resuspended in 100 μL of 1:1 chloroform:methanol.

Thin Layer Chromatography

Thin-layer chromatography (TLC) was performed by using Silica Gel 60 Aluminum Sheets (MilliporeSigma™ ; catalog no. M1055530001). 9 cm X 10 cm plates were pre-cleaned with 1:1 chloroform:methanol by developing in a Fungicrom separating TLC chamber (Fungilab™ ; catalog no. 06-815-188). After air drying the plates in a fume hood overnight, and heating with a blow dryer for 5 minutes, the lipids were spotted (30-50 µL of membrane solution) at 1.5 cm from the bottom of the plate and 1.5 cm apart from each other. A mixture of three known phospholipids from Avanti (18:1 (Δ9-Trans) PG (cat. no. 840477); 18:1 (Δ9-Trans) PE (cat. no. 850726); and 16:0 Cardiolipin (cat. no. 710333)) spotted on each TLC plate (33.33 µg each) served as a control. The plates were air dried for 30 min. The lipids in the membrane samples were then separated in the TLC chamber equilibrated in 65:25:10 chloroform:methanol:acetic acid until the solvent front reached 1 cm below the top. The plates were air dried and then stained

with 1:1 diluted molybdenum blue spray reagent (Millipore Sigma, catalog no. M1942-100ML).

After staining, TLC plates were photographed under constant lighting conditions using a smartphone, and these photographs were analyzed using ImageJ software[5]. Briefly, each image was inverted, and the integrated density of each spot was measured along with that of a blank area of the same size. The density of blank was subtracted from the spot density. The intensities were also corrected for the amount of lipids applied (based on membrane weights and volume applied to each spot), and then divided by the intensities of standard lipids on the same plate. For each sample, intensities of three major phospholipid species (PE, PG, CL) were added up which was considered to be 100 %, and the percentage of each individual phospholipid was calculated accordingly, to be plotted in graphs.

Bibliography

1. Letunic, I. & Bork, P. Interactive tree of life (iTOL) v4: recent updates and new developments. *Nucleic Acids Res.* **47,** W256–W259 (2019).
2. Dalebroux, Z. D. *et al.* Delivery of cardiolipins to the Salmonella outer membrane is necessary for survival within host tissues and virulence. *Cell Host Microbe* **17,** 441–451 (2015).
3. Rossi, R. M., Yum, L., Agaisse, H. & Payne, S. M. Cardiolipin Synthesis and Outer Membrane Localization Are Required for Shigella flexneri Virulence. *MBio* **8,** (2017).
4. Bligh, E. G. & Dyer, W. J. A rapid method of total lipid extraction and purification. *Can J Biochem Physiol* **37,** 911–917 (1959).
5. Schneider, C. A., Rasband, W. S. & Eliceiri, K. W. NIH Image to ImageJ: 25 years of image analysis. *Nat. Methods* **9,** 671–675 (2012).
